# Hematopoietic stem cells exhibit a specific ABC transporter gene expression profile clearly distinct from other stem cells

**DOI:** 10.1186/1471-2210-10-12

**Published:** 2010-09-13

**Authors:** Leilei Tang, Saskia M Bergevoet, Christian Gilissen, Theo de Witte, Joop H Jansen, Bert A van der Reijden, Reinier AP Raymakers

**Affiliations:** 1 Department of Laboratory Medicine, Laboratory of Hematology, Radboud University Nijmegen Medical Centre/Nijmegen Centre for Molecular Life Sciences, Geert Grooteplein 8, 6525GA, Nijmegen, The Netherlands; 2 Department of Human Genetics, Radboud University Nijmegen Medical Centre/Nijmegen Centre for Molecular Life Sciences, Geert Grooteplein zuid 10, 6525GA, Nijmegen, The Netherlands; 3 Department of Tumor Immunology, Radboud University Nijmegen Medical Centre/Nijmegen Centre for Molecular Life Sciences, Geert Grooteplein 26, 6525GA, Nijmegen, The Netherlands; 4 Department of Hematology and Van Creveld Clinic, University Medical Centre Utrecht, Eidelberglaan 100 3584CX, Utrecht, The Netherlands

## Abstract

**Background:**

ATP-binding cassette (ABC) transporters protect cells against unrelated (toxic) substances by pumping them across cell membranes. Earlier we showed that many ABC transporters are highly expressed in hematopoietic stem cells (HSCs) compared to more committed progenitor cells. The ABC transporter expression signature may guarantee lifelong protection of HSCs but may also preserve stem cell integrity by extrusion of agents that trigger their differentiation. Here we have studied whether non-hematopoietic stem cells (non-HSCs) exhibit a similar ABC transporter expression signature as HSCs.

**Results:**

ABC transporter expression profiles were determined in non-hematopoietic stem cells (non-HSCs) from embryonic, neonatal and adult origin as well as in various mature blood cell types. Over 11,000 individual ABC transporter expression values were generated by Taqman Low Density Arrays (TLDA) to obtain a sensitivity comparable with quantitative real-time polymerase chain reactions. We found that the vast majority of transporters are significantly higher expressed in HSCs compared to non-HSCs. Furthermore, regardless their origin, non-HSCs exhibited strikingly similar ABC transporter expression profiles that were distinct from those in HSCs. Yet, sets of transporters characteristic for different stem cell types could be identified, suggesting restricted functions in stem cell physiology. Remarkably, in HSCs we could not pinpoint any single transporter expressed at an evidently elevated level when compared to all the mature blood cell types studied.

**Conclusions:**

These findings challenge the concept that individual ABC transporters are implicated in maintaining stem cell integrity. Instead, a distinct ABC transporter expression signature may be essential for stem cell function. The high expression of specific transporters in non-HSCs and mature blood cells suggests a specialized, cell type dependent function and warrants further functional experiments to determine their exact roles in cellular (patho)physiology.

## Background

Controversy has lasted for years regarding the role of ATP-binding cassette (ABC) transporters in (cancer) stem cell integrity [1-8]. Early discovery of the relation between ABC transporters and stem cells arose from the identification of the "side population" (SP), a stem cell enriched fraction that can be isolated based on ABCG2 dependent dye efflux. SP fractions have been observed in both hematopoietic and solid tissues [2]. Later, tumor initiating cells were identified in the SP, as indicated by ABC transporter gene expression (ABCB1 or ABCG2), chemo-resistance or tumorigenicity in vivo [9,10]. Further studies showed that both in solid tumors and in hematological malignancies cancer stem cells are intrinsically resistant to a broad range of drugs and exhibit elevated expression of drug resistance related ABC transporters including ABCB1, ABCC1 and ABCG2 [11,12]. Notably, CD34+CD38- leukemic progenitors, which are ABCB1 and ABCG1 double positive [13], are able to repopulate bone marrow in irradiated NOD-SCID mice while the CD34+CD38+ counterparts that show a significantly lower expression level of ABCB1 and ABCG1 [13] have no repopulating capacities [5,14]. These findings could suggest that ABC transporters contribute to maintain stem cell properties of both normal and cancer stem cells. However, mice that lack single ABC transporters show normal steady-state hematopoiesis [7]. Also, approaches to enhance cytotoxicity during chemotherapy by inhibiting one single transporter have been hardly successful [3]. The knockdown or inhibition studies focused on one or a few transporters. As the human gene encodes for 49 ABC transporters, functional redundancy may preserve stem cell integrity and chemoresistance following abrogation of the function of one or several transporters.

Recently, we determined the ABC transporter expression signature of hematopoietic stem cells (HSCs) [13]. A large set of ABC transporters was highly expressed in both normal and malignant HSCs compared to more committed progenitors [13]. Thus, the absence of obvious (stem cell) phenotypes following disruption of one or more ABC transporters may be caused by relatively high expression of other transporters. In this study we aimed to investigate whether other non-related stem cells exert a similar ABC transporter signature as HSCs and profiled transporter expression in human non-HSCs including unrestricted somatic stem cells (USSCs), mesenchymal stem cells (MSCs), embryonic stem cells (ESCs) and multipotent adult progenitor cells (MAPCs). To compare the expression signatures with differentiated cells we also analyzed human mature blood cell types. To obtain maximal detection sensitivity over a large range of expression levels, Taqman Low Density Arrays (TLDA) were employed to determine gene expression [13,15].

## Methods

### Human non-HSCs and mature blood cells

Human cord blood derived USSCs (n = 6) were generated and differentiated towards the osteogenic lineage as described [16,17]. Mature human blood cells were obtained from normal bone marrow or peripheral blood by Ficoll gradient centrifugation (granulocytes) or by fluorescence activated cell sorting with specific cell surface markers to gain a purity above 95%: CD3+ (T cells), CD56+CD3- (NK cells), CD14+ (monocytes), CD71+ (erythroid progenitors) as described previously [18,19]. Total mRNA was subsequently isolated using RNABee (Bio-Connect BV, the Netherlands). mRNA of human MSCs derived from adult adipose tissue (n = 3) and bone marrow (n = 4) was kindly provided by Dr. E. Piek, Department of Applied Biology and Dr. B. Jansen, Tumor Immunology Laboratory, Radboud University Nijmegen Medical Center, the Netherlands [20]. mRNA of human bone marrow derived MAPC lines (n = 3) was provided by Dr. C. Verfaillie, Leuven. The mRNA of three hESC cell lines (HUES1, HES2, and HES3), originating from Douglas Melton's Lab (HUES1) and ES Cell International, Singapore http://www.escellinternational.com (HES2 and HES3), were kindly provided by Prof. Dr. C. Mummery, Department of Anatomy and Embryology, Leiden University Medical Centre, the Netherlands. Transporter expression profiles in HSC samples (n = 11) that we observed earlier [13] were taken as reference for comparisons with the profiles in non-HSCs and mature blood cell types.

### TaqMan Low Density Array

First strand cDNA of the mRNA samples indicated above was synthesized using reverse transcriptase as described (Invitrogen) [19]. Gene expression profiles were assessed by quantitative real-time RT-PCR using micro fluidic cards (Taqman Low Density Arrays, TLDA, Applied Biosystem), covering 45 transmembrane ABC transporters and three housekeeping genes (GAPDH, HPRT1 and HMBS) as described earlier [13]. The expression levels were calculated relative to GAPDH (Additional file 1: TLDA data table on all the studied cell types).

### Statistical analyses

Over 11,000 ABC transporter expression values were generated by TLDA. Some transporter genes were expressed at very low levels around the detection limit, introducing unwanted disturbance in statistical analyses. To avoid this, the highest mean expression level in the HSCs (6.65E-02, observed for ABCA2 in HSCs samples) was divided by 1000 (6.65324E-05) and used as background level. Expression values below the background level were replaced by this threshold. In this way the noise caused by the variation in extremely low values was abolished. Principal component analysis (PCA) [21,22] was performed using Partek Genomic Suite 6.4 to identify outliers in our sample sets and to investigate similarities of samples based upon the cell types. Unsupervised hierarchical clustering was performed by using Partek Genomic Suite 6.4 to visualize genes defining different cell types and to determine the similarity/distance between the studied cell types. We used the Pearson dissimilarity as a distance measure. Differential expression was examined by a non-parametric Mann-Whitney U-test in Partek Genomic Suite 6.4. Genes with P-values < 0.05 after multiple testing correction by bootstrapping were deemed significantly differentially expressed.

## Results

### Non-HSCs show striking similarities in ABC transporter expression profiles clearly distinct from HSCs

**Figure 1:**
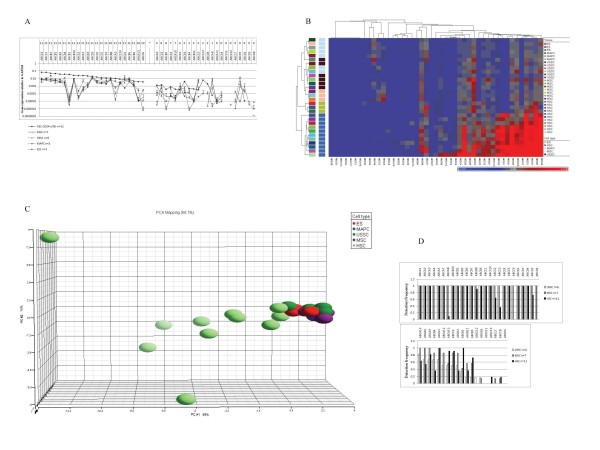
**Non-HSCs show striking similarity in ABC transporter expression profiles clearly distinct from HSCs**. (A) TLDA expression data plot. The expression levels relative to GAPDH are shown in log-scale, plotted against the ABC transporter genes ranked in descending order by expression levels in 11 CD34+38- HSC samples. The expression profile of CD34+38- HSCs here serves as reference [13]. The transporters are further divided into two groups (left and right) according to the detection frequency (whether above 10/11) in the HSCs. Count of detectable samples out of the 11 reference HSC samples is indicated on the top of the figure for each transporter. Transporters genes in the right part are ranked first by detection frequency and then by the expression level. The error bars show the standard deviations. In this plot the background threshold applied for unsupervised hierarchical clustering and PCA was not used. (B) Unsupervised hierarchical clustering allowing a separation between HSCs and non-HSCs based on ABC transporter expression profiles. Red and blue indicate high and low expression, respectively. (C) PCA of HSCs and non-HSCs based on ABC transporter expression profiles verified the resemblance among non-HSCs. This PCA mapping represents 68.1% of gene expression information. (D) Detection frequency of each transporter gene in HSCs, MSCs and USSCs. Note that some transporters are detected in all studied USSCs and MSCs but not in HSCs (e.g. ABCA4 and ABCC9).

Earlier we showed that a broad range of ABC transporters was highly expressed in HSCs compared to more committed progenitor cells [13]. To determine the relevance of this finding, we measured by TLDA transporter expression profiles in non-HSCs including USSCs, MSCs, MAPCs and ESCs. Striking to note is that non-HSCs, regardless their origin, exhibited a remarkable similarity in transporter expression clearly distinct from that observed in HSCs (Fig. 1 A). Unsupervised hierarchical cluster analysis confirmed the strong similarity among non-HSCs. The 11 HSC samples clustered together, with a distinguishable distance to other stem cell types (Fig. 1 B). MAPCs and ESCs also clustered in separate groups while USSCs and MSCs fell into one and the same group, suggesting that MSCs and USSCs are closely related (Fig. 1 B). Principal component analysis (PCA) verified the resemblance among non-HSCs (Fig.1 C). Among the HSC samples a larger relative distribution was observed as compared to non-HSCs, indicating that differences within HSC samples are greater than those among other stem cells. Another important observation is that most transporters showed higher expression in HSCs than in other stem cell types, except for ABCA1, ABCB6, ABCB8 and ABCD3, which were similarly expressed in both HSCs and non-HSCs (Fig. 1 A). The mean expression values of ABCB1 and ABCG1 were more than 1000-fold higher in HSCs while the other genes about 10-fold higher. Interestingly, some transporter genes, such as ABCA4, ABCA8, ABCC9 and ABCG4, were scarcely detectable in HSC samples whereas they were consistently detected in USSCs and MSCs (Fig.1 A and 1D).

To identify genes representative for each stem cell type, we performed Mann-Whitney U-tests. Sixteen genes defined HSCs from non-HSCs (Additional file 2: Genes defining HSCs from other stem cells). Amongst these genes some are known to be implicated in hematopoiesis, such as ABCB1 and ABCG1 [2,4,5,23]. Notably, ABCB1 is the gene to distinguish HSCs with the highest fold difference in median value (1.41E + 04 fold). Nevertheless, other transporter genes with no described hematopoietic functions, like ABCA3, ABCA5, ABCA7 and ABCD4, also discriminate HSCs from non-HSCs.

Despite the strong similarity regarding transporter expression in non-HSCs, distinct sets of ABC transporter genes could be identified typifying each non-HSC source by Mann-Whitney U-tests (Additional file 3: Combination of ABC transporter genes unique for each subgroup of cells). Interestingly, some transporters not reportedly related to stem cell functions were significantly higher expressed in ESCs compared to other non-HSCs, such as ABCA3, ABCA7 and ABCC8. ABCG2 was differentially higher expressed in the ESC samples we studied and the expression of ABCA8 and ABCC9 was about 1000-fold lower in ESCs than in other non-HSCs. MAPCs were highlighted by ABCA13 with an expression level about 60-fold higher than in other non-HSC sources. MSCs also showed differential expression of several transporters while for USSCs only one differentially expressed transporter (ABCD4) was observed (Additional file 3). In summary, non-HSCs exhibited strikingly similar ABC expression profiles remarkably distinct from those in HSCs and HSC exhibited overall higher expression levels compared to non-HSCs. Characteristic sets of transporters in HSCs and different non-HSCs were identified, suggesting that specific combinations of transporters are important for the physiology of non-related stem cells.

### ABC transporters are not down modulated following osteogenic differentiation of USSCs

**Figure 2:**
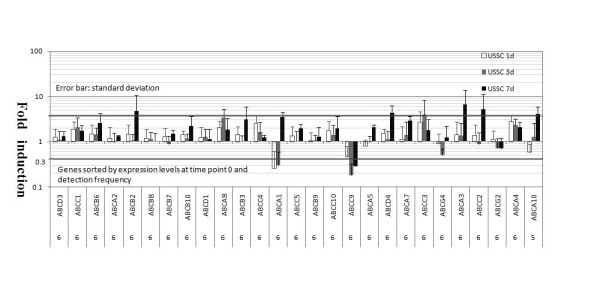
**ABC transporters are not down modulated following osteogenic differentiation of USSCs**. The expression levels on day 0 are set at 1 and the fold difference in expression at other time points is given on day 1, day 3 and day 7. Significant threshold is 3-fold induction or downregulation and is indicated by the grey lines. Detection count of each gene in 6 USSC samples is indicated on the bottom of the figure for each transporter. Only transporters with detection frequency above 5/6 in USSCs are included.

We have shown that ABC transporter expression was down modulated during the differentiation of both normal and leukemic HSCs [13]. Besides, we recently observed that some miRNAs were down regulated following osteogenic differentiation of USSCs [17]. These findings prompted us to study whether ABC transporter expression decreases following USSC differentiation. However, transporter profiling by TLDA did not reveal broad down regulation as observed in the initial steps of HSC maturation. On the contrary, some transporters were even induced (Fig. 2). This suggests that ABC transporters may serve a different function in USSCs compared to HSCs.

### Mature blood cells exhibit higher expression of specific transporters compared to HSCs

**Figure 3:**
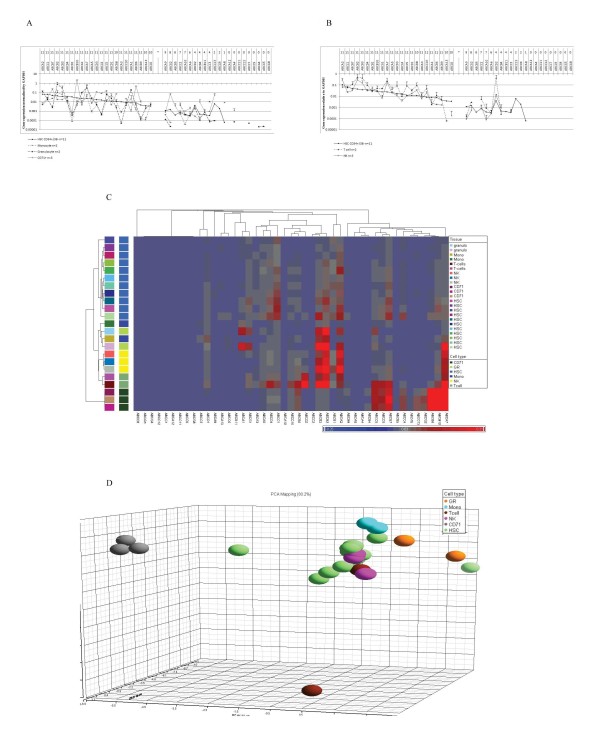
**Mature blood cells exhibit higher expression of specific transporters compared to HSCs**. (A) (B) TLDA expression data plot. The layout of the plot is as described in the legend of figure 1 A. (C) Unsupervised hierarchical clustering of HSCs and mature blood cell types based on ABC transporter expression profiles. (D) PCA of HSCs and mature blood cell types based on ABC transporter expression profiles. This PCA mapping represents 80.2% of gene expression information.

The substantial expression of a broad range of ABC transporters compared to non-HSCs and committed progenitors may indicate that they play an important role in HSC biology. The implication of these transporters in HSC maintenance may be also inferred by their down regulation during early steps in HSC differentiation [13]. To study this issue in more detail we measured transporter profiles in various mature blood cell types including monocytes, granulocytes, CD71+ erythroid progenitors, T cells and NK cells in order to determine whether the high expression is unique to HSCs. Unexpectedly, most transporters were expressed at a higher level in at least one of the mature blood cell types rather than in HSCs (Fig. 3A and 3B). For instance, in T cells, NK cells and granulocytes the expression of ABCA7, ABCB2 and ABCB3 was above 10-fold higher than in HSCs. The expression of ABCD2 in T cells was evidently elevated in comparison with any other blood cell fraction. ABCB1, a gene with widely reported relevance to stem cells, exhibited in NK cells an expression level exceeding that in HSCs. Cholesterol transporter ABCA1 and lipid transporter ABCA2 were evidently higher expressed in granulocytes and NK cells, respectively, compared to HSCs. Likewise, the expression of ABCB6 and ABCB10 and ABCG2 in CD71+ erythroid progenitors was more than 40-fold above that in HSCs. The highest expression level of all the studied cell types was observed for ABCB10 in CD71+ erythroid progenitors. Unlike other transporters, ABCA13 and ABCC1 showed slightly higher expression in HSC samples compared to all mature blood cell types (less than 2-fold higher compared to granulocytes and T cells) (Fig. 3A and 3B). Consistent with the TLDA plots (Fig 3AB), unsupervised hierarchical clustering presented higher expression levels mostly in more mature blood cell samples (Fig. 3C). HSC samples fell into a distinct group apart from mature blood cell types in both unsupervised clustering and PCA. Characteristic expression patterns evidenced by separated clusters were observed for each different mature blood cell fraction except for monocytes and granulocytes, which fell into one and the same group in unsupervised hierarchical cluster analysis (Fig. 3C and 3D). Of note, the CD71+ erythroid progenitor samples constituted a tight cluster in PCA, identifying these samples as a highly homogenous and specialized cell type (Fig. 3D). Differential gene expression through subgroups of mature blood cell fractions was determined by a Mann-Whitney U-test as indicated in additional file 3. Significant differences were identified in NK cells and CD71+ erythroid progenitors. ABCB1 was found to represent NK cells; so were ABCB6, ABCB10 and ABCG2 representative for erythroid progenitors.

## Discussion

A remarkable finding of this study is that non-HSCs from adult, neonatal or embryonic origins exhibit a very similar ABC transporter expression profile that is clearly different from HSCs (Fig. 1 A, B and 1C). Apparently HSCs rely on a different repertoire of transporters whereas for other stem cells it is more similar. Three separate clusters can be seen in Fig. 1 B for MAPCs, ESCs and USSCs/MSCs, despite the strong resemblance regarding transporter expression in non-HSCs. Although USSCs differ from MSCs with respect to telomere length, immunophenotype and differentiation potential [16], they fell into one and the same cluster in unsupervised hierarchical cluster analysis based on transporter expression profiles (Fig. 1 B). This indicates a close similarity between USSCs and MSCs. Nevertheless, comparison of profiles among non-HSCs revealed differential transporter expression, especially for MAPCs and ESCs (Additional file 3). ABCA13 was representative for MAPCs by an expression level about 60-fold higher compared to other non-HSC sources. ABCA13 is expressed in human hippocampus and cortex and rare variants in this gene have been associated with neurological disorders [24]. Since ABCA family members share a cholesterol efflux related C-terminal motif, the function of ABCA13 might be lipid related as well [25]. It may explain a role for ABCA13 in neurological disorders since lipid shuttling across cell membranes is crucial to intracellular signalling pathways and neurotransmitter functions. This might imply that MAPCs rely more on lipid metabolic pathways than other non-HSCs do. Of note, ABCG2, a transporter of intense debate regarding its expression in human ESCs [8,26], was expressed at significantly higher levels in the ESC samples studied here compared to other non-HSCs (Fig. 1). This is in agreement with the study of Sarkadi et al [26]. As a principal multidrug resistant gene [1,2,4,27-31], ABCG2 can potentially protect undifferentiated ESCs against the damage caused by toxins or hypoxia. Specific roles in human disorders or physiological functions for several other transporters that are differentially expressed through non-HSC samples (Additional file 3) have been reported [32-36]. Further research on their exact function in stem cells is warranted.

Another important finding here is that out of the 23 most frequently detected and highly expressed transporters in HSCs, the majority showed lower expression in non-HSCs (Fig. 1A). Mann-Whitney U-test revealed 16 transporters to be expressed at significantly higher levels in HSCs compared to other stem cells (Additional file 2). Out of these 16 transporters, the expression of ABCB1 and ABCG1 was above 1000-fold higher in HSCs. The role of ABCB1 in bone marrow repopulation capacity and chemoresistance has been intensively studied and widely reported [1,4,5,28,37,38]. ABCG1 has been shown to regulate proliferation of hematopoietic stem cells through high-density lipoprotein [23,39]. ABCG2, one of the major multidrug transporters [1,2,4,27-31], is not consistently detectable in HSCs, which is in agreement with a recent report showing that ABCG2 expression is not correlated to hematopoietic progenitor function [6]. On the other hand, some differentially expressed genes (above 10-fold higher) have not been reported to have immediate relevance to hematopoiesis, such as ABCA3, ABCA5, ABCA7 and ABCD4. These genes might be involved in lipid transport or intracellular trafficking of peroxisomes or lysosomes [32-34,40-42]. Lipid redistribution is required for polarization of HSCs, which is essential to initiate migration, hence lipid transporters (such as ABCA3 and ABCA7) may potentially influence HSC migration [31]. A few transporters, including ABCA1, ABCB6, ABCB8 and ABCD3, were similarly expressed in both HSCs and non-HSCs but still at a lower level compared to mature blood cell types. Notably, a set of transporters was consistently detected in MSCs and USSCs but scarcely detectable in HSCs, including ABCA4, ABCA8, ABCC9 and ABCG4 (Fig. 1 D). ABCA4, ABCA8 along with ABCG4 might play a role in brain lipid transport [42] and ABCC9 constitutes the regulatory subunit of an ATP-sensitive potassium channel [43,44]. These four transporters were not significantly expressed in mature blood cell types either, representing a specific signature of non-HSCs.

To further study the relevance of the finding that a broad range of transporters exhibited higher expression levels in HSCs compared to non-HSCs we analyzed mature blood cell types. It was observed that only ABCA13 and ABCC1 were slightly higher expressed in HSCs than in mature blood cell types. As discussed above, ABCA13 might play a role in lipid redistribution [24,25,31]. As HSC polarization is accompanied by redistribution of transmembrane lipid rafts and a polarized morphology is required for cells to initiate migration [31], it will be interesting to investigate whether ABCA13 contributes to HSC migration. ABCC1 is known to be involved in chemoresistance of hematological cancer stem cells [11,12], however it is not obviously down regulated throughout differentiation, questioning its contribution to stem cell integrity. Actually most transporters showed evidently higher expression in developed blood cell types (Fig. 3A,B and 3C), challenging the concept that individual transporters may function in maintaining stem cell integrity [1,2,4,5]. For instance, outstanding high expression levels were observed for ABCB2 and ABCB3 in granulocytes, T cells and NK cells (Fig. 3A and 3B). ABCB2 (TAP1) and ABCB3 (TAP2) belong to the transporter associated with antigen processing (TAP) family, which is crucial for the adaptive immune system. TAP translocates proteasomal degradation products into the endoplasmic reticulum, in order to present them to the major histocompatibility complex (MHC) I molecules [45]. This suggests an association of the high expression of ABCB2 and ABCB3 with their specific immunological function in T cells and granulocytes. In CD71+ erythroid progenitors the expression of ABCB6, ABCB10 and ABCG2 was extremely high in comparison with HSCs and other mature blood cell types. These transporters have been implicated in porphyrin transport and/or heme biosynthesis, which explains their high expression in haemoglobin synthesizing cells[46-51]. Given that mature blood cell types exhibit higher transporter expression compared to HSCs, it will be important to compare transporter expression signatures in tissues derived from non-HSCs.

## Conclusions

Here we identified specific ABC transporter expression signature in non-HSCs compared to HSCs. Four transporters, ABCA4, ABCA8, ABCC9 and ABCG4, are of special interest since they are expressed in non-HSCs but hardly detectable in HSCs or mature blood cells. The high expression of several transporters in committed blood cells challenges the concept that individual ABC transporters may maintain stem cell integrity by protecting them against xenobiotics [1,2,4,5]. Instead, a distinct ABC transporter expression signature might be essential for stem cell function rather than overexpression of single transporters. These findings warrant further studies on specific transporters in different cell types.

## List of abbreviations

ABC transporters: ATP-binding cassette transporters; HSCs: Hematopoietic stem cells; Non-HSCs: Non-hematopoietic stem cells; TLDA: Taqman Low Density Arrays; USSCs: Unrestricted somatic stem cells; MSCs: Mesenchymal stem cells; ESCs: Embryonic stem cells; MAPCs: Multipotent adult progenitor cells

## Authors' contributions

LT participated in micofluidic card experiments, performed the data analysis and drafted the manuscript. SB carried out the micofluidic card experiments and participated in cell line material collection. CG performed the statistical analysis. TW participated in the design of the study. JJ participated in the design of the study. BR conceived of the study, participated in its design and coordination and helped to draft the manuscript. RR conceived of the study, and participated in its design and coordination and helped to draft the manuscript. All authors have read and approved the final manuscript.

## Authors' information

The authors report no conflict of interest in connection with this manuscript.

## Supplementary Material

Additional file 1 **TLDA data table on all the studied cell types**. Click here for file

Additional file 2 **Genes defining HSCs from other stem cells**. Click here for file

Additional file 3 **Combination of ABC transporter genes unique for each subgroup of cells**. Click here for file
